# Human Liver-Derived Extracellular Matrix for the Culture of Distinct Human Primary Liver Cells

**DOI:** 10.3390/cells9061357

**Published:** 2020-05-30

**Authors:** Niki Alevra Sarika, Valéry L. Payen, Maximilien Fléron, Joachim Ravau, Davide Brusa, Mustapha Najimi, Edwin De Pauw, Gauthier Eppe, Gabriel Mazzucchelli, Etienne M. Sokal, Anne des Rieux, Adil El Taghdouini

**Affiliations:** 1 Laboratory of Pediatric Hepatology and Cell Therapy (PEDI), Institute of Experimental and Clinical Research (IREC), Université catholique de Louvain (UCLouvain), Avenue Mounier 52 Box B1.52.03, 1200 Brussels, Belgium; niki.alevra@uclouvain.be (N.A.S.); valery.payen@uclouvain.be (V.L.P.); joachim.ravau@uclouvain.be (J.R.); mustapha.najimi@uclouvain.be (M.N.); 2 Laboratory of Advanced Drug Delivery and Biomaterials (ADDB), Louvain Drug Research Institute (LDRI), Université catholique de Louvain (UCLouvain), Avenue Mounier 73 Box B1.73.12, 1200 Brussels, Belgium; 3 GIGA-Proteomics Facility, University of Liège (ULiège), Allée du six août 11, 4000 Liège, Belgium; m.fleron@uliege.be; 4 Mass Spectrometry Laboratory (MSLab), MolSys Research Unit, University of Liège (ULiège), Allée du six août 11, 4000 Liège, Belgium; e.depauw@uliege.be (E.D.P.); g.eppe@uliege.be (G.E.); gabriel.mazzucchelli@uliege.be (G.M.); 5 Flow Cytometry Technological Platform, Institute of Experimental and Clinical Research (IREC), Université Catholique de Louvain (UCLouvain), Avenue Hippocrate 55 Box B1.55.20, 1200 Brussels, Belgium; davide.brusa@uclouvain.be

**Keywords:** liver, primary cells, extracellular matrix, decellularization, proteomics

## Abstract

The lack of robust methods to preserve, purify and in vitro maintain the phenotype of the human liver’s highly specialized parenchymal and non-parenchymal cell types importantly hampers their exploitation for the development of research and clinical applications. There is in this regard a growing interest in the use of tissue-specific extracellular matrix (ECM) to provide cells with an in vitro environment that more closely resembles that of the native tissue. In the present study, we have developed a method that allows for the isolation and downstream application of the human liver’s main cell types from cryopreserved material. We also isolated and solubilized human liver ECM (HL-ECM), analyzed its peptidomic and proteomic composition by mass spectrometry and evaluated its interest for the culture of distinct primary human liver cells. Our analysis of the HL-ECM revealed proteomic diversity, type 1 collagen abundance and partial loss of integrity following solubilization. Solubilized HL-ECM was evaluated either as a coating or as a medium supplement for the culture of human primary hepatocytes, hepatic stellate cells and liver sinusoidal endothelial cells. Whereas the solubilized HL-ECM was suitable for cell culture, its impact on the phenotype and/or functionality of the human liver cells was limited. Our study provides a first detailed characterization of solubilized HL-ECM and a first report of its influence on the culture of distinct human primary liver cells.

## 1. Introduction

The liver fulfills a myriad of vital functions due to its complex assembly of highly specialized parenchymal and non-parenchymal liver cells. The liver parenchymal fraction (PF) consists mainly of hepatocytes (HEPs), which represent 60% of the total number of liver cells [1]. The non-parenchymal fraction (NPF), in contrast, is composed of multiple, less abundant cell populations, including the vitamin A and antigen presenting hepatic stellate cells (HSCs), the fenestrated liver sinusoidal endothelial cells (LSECs) and the Kupffer cells, the largest population of tissue macrophages (MPs). Altogether, parenchymal and non-parenchymal cells orchestrate liver functions, maintain liver tissue homeostasis and play a central role in the liver regeneration process [2,3,4,5]. Therefore, there is a growing interest in new, standardized methods to isolate, expand and exploit the human liver’s distinct cell types for the development of clinical (e.g., cell therapies and engineered tissues) and research applications (e.g., toxicology and drug discovery tools).

A number of hurdles however significantly limit the development of such applications. Besides the scarce and highly unpredictable availability of human liver tissue, the lack of efficient methods to produce human liver cell preparations of high quality and purity is an additional limiting factor. Whereas the cryopreservation of freshly isolated HEPs has been the subject of numerous studies [6], the cryopreservation of the NPF, prior to and in view of its further processing for the purification of the different non-parenchymal liver cell types, has been poorly studied. Another important hurdle constitutes the rapid loss of the specific phenotype of human primary liver cells in vitro. It is indeed well-known that, upon in vitro culture, HEPs rapidly lose their polarity and metabolic properties [7,8], HSCs undergo an activation process during which they rapidly lose their retinoid-containing lipid droplets and acquire a fibrogenic, myofibroblast-like phenotype [9,10], and LSECs lose their fenestrations and sinusoidal-specific transcriptomic signature [11,12]. There is in this regard a growing interest in the use of liver ECM derivatives as a coating, supplement or substrate to provide liver cells with an environment that presents structural, mechanical and biochemical characteristics closer to their native microenvironment. However, studies so far have primarily focused on evaluating the impact of liver ECM derivatives on the culture of immortalized cell lines or animal-derived cells and have collectively returned ambiguous results [13,14,15,16,17,18]. To our knowledge, the composition of human liver-specific ECM (HL-ECM) and its potential impact on the function and phenotype of the main human liver cell populations has not yet been investigated.

In the present study, we developed and validated a procedure to simultaneously purify distinct human liver cell populations from cryopreserved whole liver cell suspensions. We also produced and for the first time characterized the proteomic and peptidomic content of solubilized HL-ECM prior to evaluating its impact on the phenotype and functional properties of purified primary human liver cells in vitro.

## 2. Materials and Methods

### 2.1. Sourcing of Fresh Human Liver Tissue

Whole human livers not suitable for transplantation and human liver fragments were provided by the Hepatic Biobank of the Cliniques Universitaires Saint-Luc (CUSL). All protocols and experiments were approved by the ethical committees of CUSL and by the Faculty of Medicine of UCLouvain (Agreements 2015/03NOV/585 and 2019/04MAR/100). In accordance with Belgian law, the tissue donors and/or their families received the necessary information and provided active or passive consent for the use of human residual material for research purposes. The clinical characteristics, age and sex of the donors are listed in Table S1 (Supplementary Materials).

### 2.2. Isolation, Cryopreservation and Purification of Human Primary Liver Cells

The liver cell suspensions were obtained from fresh, whole livers or whole liver lobes by a classical two-step perfusion method, as described before [19,20]. Briefly, the liver tissue was sequentially perfused with 37 °C prewarmed Earle’s balanced salt solutions (Lonza, Basel, Switzerland) containing 440 μM of calcium-chelating agent EGTA (Sigma-Aldrich, St. Louis, MO, USA) and 1400 UI/L of collagenase (Sigma-Aldrich). The resulting total liver cell suspension was further separated into the PF and the NPF by two low-speed centrifugation steps (36 g and 56 g, 10 min each at 4 °C). Both fractions were cryopreserved at a density of 10–20 million viable cells/mL in IGL1-solution (Institut Georges Lopez, Lissieu, France) [21], supplemented with 15% Hibumine (Baxter, Vienna, Austria) and 10% DMSO (WAK-Chemie Medical, Steinbach, Germany). Progressive cooling was performed at 1 °C/min from 0 to −40 °C and at 2 °C/min from −40 to −80 °C in a Nicool Freezal (Air Liquide, Paris, France). Cell fractions with a viability <70%, evaluated by Trypan blue dye exclusion test (GE Healthcare Life Sciences, IL, USA), were excluded. All cell purifications described in this study were performed starting from these cryopreserved PF and NPF.

HEPs were obtained by thawing the PF in cryopreserved hepatocyte recovery medium (Gibco, Waltham, MA, USA) according to the manufacturer’s instructions. HSCs were isolated from the NPF as previously described by centrifugation in a Nycodenz (Nyegaard and Co., Oslo, Norway) or Optiprep (Axis-Shield, Dundee, United Kingdom) gradient of density [10,22]. HSCs were collected at the upper interface of the gradient. Liver MPs and (LS)ECs were purified from the NPF according to their selective expression of CD14 and CD146. In brief, cryopreserved NPF was thawed in SOPP-SS 4% solution (CAF-DCF, Brussels, Belgium) supplemented with 2.5 g/L glucose (STEROP, Brussels, Belgium), 10 mM NaHCO_3_ (STEROP, Brussels, Belgium) and 200 UI/L heparin (Leo Pharma, Copenhagen, Denmark), filtered on a 70 μm mesh (Corning, NY, USA) and (i) separated according to CD14 and CD146 expression by fluorescence-activated cell sorting (FACS, FACSAria III, BD Biosciences, Franklin Lakes, NJ, USA), or (ii) successively separated according to CD146 and then CD14 expression by magnetic beads-activated cell sorting (MACS) on LS columns (Miltenyi Biotec, Bergisch Gladbach, Germany) according to the manufacturer’s instructions. Microbeads conjugated with an anti-human CD14 and with an anti-human CD146 and human FcR blocking reagent (Miltenyi Biotec) were used for MACS. FITC-conjugated anti-human CD14 (clone REA599) and PE-conjugated anti-human CD146 (clone 541-10B2) (Miltenyi Biotec) were used for FACS. At least 20,000 events were acquired for FACS analysis.

### 2.3. Human Liver Tissue Decellularization and Preparation of Solubilized HL-ECM

Cryopreserved healthy liver fragments (non-inflamed, non-fibrotic) from four donors were decellularized as previously described [13,17,23] with some modifications (Figure S1A). First, liver fragments were rinsed and cut into 0.5 cm^3^ pieces and frozen at −80 °C. All tissue decellularization steps were performed under a laminar flow using 0.22 µm-filtered solutions and autoclaved instruments and glass. The liver pieces were thawed, washed 3 times in deionized water, gently massaged and successively incubated (i) in a pH 8-adjusted solution containing 0.02% trypsin (Gibco) and 0.005% EDTA (AppliChem, Darmstadt, Germany) at 37 °C for 4 h and (ii) in 3% Triton X 100 (Carl Roth, Karlsruhe, Germany) for 24 h at room temperature (RT). The last two steps were repeated twice. Next, decellularized liver pieces were treated with 0.5 mg/mL DNase I (Roche) in a pH 7.6-buffered solution (10 mM TrisHCl, 5 mM EDTA) and disinfected with a 0.01% peracetic acid solution for 2 h each. The decellularized liver pieces were subsequently washed extensively in PBS and in deionized water. Finally, HL-ECM was freeze-dried.

HL-ECM was solubilized by pepsin digestion as previously described [24]. Briefly, fragments of freeze-dried HL-ECM were incubated at a concentration of 10 mg/mL in a solution containing 1 mg/mL of pepsin and 0.01 M HCl, and stirred for 48 h at RT. Undigested ECM was pelleted while the supernatant was stored at −20 °C. Before use, pepsin digests were thawed on ice, pH was neutralized and salt concentration was balanced.

For quality control of the decellularization, DNA was extracted from lyophilized HL-ECM and solubilized HL-ECM using the QIAamp DNA Mini kit (Qiagen, Hilden, Germany) and quantified with CyQUANT cell proliferation assay kit (Invitrogen, Waltham, MA, USA) according to the manufacturers’ instructions. After quantifying (Pierce BCA assay kit, Thermo Scientific, Waltham, MA, USA) and adjusting the protein concentration to the same value for each donor-derived solubilized HL-ECM (1 mg/mL for medium supplementation or 100 µg/mL for coating), we pooled the solubilized HL-ECM from four donors in equal proportions to attenuate variations.

For the staining of collagen fibers (Figure S1B), lyophilized HL-ECM was embedded in OCT and cut as 10 μm-thick cryosections before fixation during 10 min in 4% formaldehyde and staining with Sirius Red. Solubilized HL-ECM (1 mg/mL) was fixed overnight in 4% formaldehyde, successively incubated with 30% and 10% sucrose solutions and embedded in OCT before cutting as 10 μm-thick cryosections and staining with Sirius Red.

### 2.4. Proteomic Analysis of Lyophilized HL-ECM and Solubilized HL-ECM

Unless stated otherwise, all the chemicals used were purchased from Sigma-Aldrich.

#### 2.4.1. Peptide Fraction Analysis

5 mg of lyophilized HL-ECM or the equivalent of 1 mg of protein from solubilized HL-ECM were precipitated in 80% acetonitrile (ACN)/20% H_2_O at a final concentration of 10 mg protein/mL for lyophilized HL-ECM or 1.2 mg protein/mL for solubilized HL-ECM. Samples were then sonicated 3 × 15 s in an ice water bath (Branson, Danbury, CT, USA) and heated 3 min at 99 °C, each step repeated twice, before 16 h of incubation at 4 °C under agitation (600 RPM). The samples were then centrifuged at 16,000× *g* for 5 min to collect the supernatants. Supernatants containing peptides were lyophilized and subsequently solubilized in 50 mM NH_4_HCO_3_ and quantified using the Micro BCA protein assay kit (Thermo Scientific) according to the manufacturer’s instructions. Then, peptide samples were reduced for 40 min with 10 mM dithiothreitol (DTT) at 56 °C, alkylated during 30 min with 20 mM iodoacetamide at RT and further reduced for 10 min with 21 mM DTT (final concentration) at RT. The peptide samples were then acidified with trifluoroacetic acid (TFA, 0.1% final), desalted using C18 ZipTip (Millipore, Bedford, MA, USA) and freeze-dried. Peptides were solubilized to reach 1 µg/9 µL 0.1% TFA just prior to mass spectrometry (MS) analyses.

#### 2.4.2. Protein Fraction Analysis

0.5 mg of lyophilized HL-ECM or the equivalent of 0.2 mg of protein from solubilized HL-ECM were first precipitated during 1 h at −20 °C in acetone/trichloroacetic acid (TCA) to reach a sample/acetone/TCA volume ratio of 1/8/1). After precipitation, samples were centrifuged at 16,000× *g* during 5 min at 4 °C. The proteins forming the pellets were washed 3× with ice-cold acetone by centrifugation. The resulting pellets were then suspended in 50 mM NH_4_HCO_3_, sonicated 3 × 15 s in an ice water bath and heated 3 min at 99 °C (each step repeated twice), and subsequently quantified using the RC-DC Protein Assay Kit (Bio-Rad, Irvine, CA, USA) according to the manufacturer’s instructions. After this, the samples were reduced for 40 min with 10 mM DTT at 56 °C, alkylated during 30 min with 20 mM iodoacetamide at RT. Then, the protein samples were subsequently digested using a multienzyme digestion step as previously described [25]. The resulting fractioned proteins were then acidified with TFA at 0.1% final concentration (Acros Organics), desalted using the C18 ZipTip and freeze-dried. Peptides were solubilized to reach 1 µg/9 µL 0.1% TFA just prior to the MS analyses.

Samples corresponding to the peptide and protein fractions were analyzed using an ACQUITY UPLC M-Class system (Waters Corporation, Milford, MA, USA) coupled to a Q Exactive Hybrid Quadrupole-Orbitrap mass spectrometer (Thermo Scientific) in the electrospray positive ion mode. An amount of 1 µg was injected per sample replicate. The 1D-UPLC system configuration was composed of a nanoEase M/Z Symmetry C18 and a nanoEase M/Z HSS C18 T3 as trap and analytical columns, respectively (Waters Corporation). The samples were loaded at 20 μL/min on the trap column in 100% solvent A (0.1% formic acid in water) during 3 min and subsequently separated on the analytical column (flow rate 600 nL/min, solvent A and solvent B (0.1% formic acid in acetonitrile), linear gradient 0 min 98% A, 5 min 93% A, 135 min 70% A and 150 min 60% A). The remaining 30 min were used for cleaning and re-equilibration steps. The total run time was 180 min. The LC eluent was directly electrosprayed from the analytical column at 2.1 kV. A TopN-MSMS method was used with *n* set to 12, meaning that the spectrometer acquires the full MS spectrum, selects the 12 most intense peaks in this spectrum (singly charged precursors excluded) and acquires the full MS2 spectrum of each of these 12 compounds. The parameters for MS spectrum acquisition were: mass ranged from 400 to 1750 *m*/*z*, resolution of 70,000 and an automatic gain control (AGC) target of 106 or maximum injection time of 50 ms. The parameters for MS2 spectrum acquisition were: isolation window of 2.0 *m*/*z*, collision energy of 25, resolution of 17,500, AGC target of 105 or maximum injection time of 50 ms. MS/MS spectra were compared to the human protein database (Uniprot_HomoSapiens) using Proteome Discoverer 2.1.1.21 software (Thermo Scientific). All protein identifications required detection of 1 unique peptide per protein. When available, the relative protein abundance was determined in the protein fraction according to the average area of the 3 most abundant unique peptides [26].

### 2.5. Electrophoresis

For 1D SDS-PAGE, 10 µg of proteins were precipitated with the 2D-Clean-Up kit (GE Healthcare) and subsequently dissolved in Laemmli buffer (65 mM Tris-HCl pH 6.8, 20% glycerol, 2% SDS, 350 mM DTT and trace of bromophenol blue), boiled for 5 min at 95 °C and loaded on a NuPAGETM 4–12% Bis-Tris Gel (Invitrogen). The gel was run in the MOPS SDS Running Buffer (Invitrogen) and stained with Coomassie Brilliant Blue.

For the 2D SDS-PAGE, after the 2D-Clean-Up kit (GE Healthcare), 15 µg of proteins were dissolved in 7 M urea, 2 M thiourea, 1.5% ASB-14, 1.5% CHAPS, 20 mM Tris-HCl pH 8.5, destreak reagent 12.5 μL/mL, 0.5% IPG buffer and a trace of bromophenol blue. The diluted samples were loaded on an immobilized pH gradient strip (Immobiline DryStrip pH 3–10 NL 7 cm, GE Healthcare) through passive rehydration (overnight). The isoelectric focusing step was performed on an Ettan IPGphor 3 (GE Healthcare) and consisted of a 1 h step at 150 V. Voltage was then increased from 150 to 1000 V in 1 h and then increased from 1000 to 2000 V during 1h. A constant voltage of 2000 V was then applied until reaching a final amount of 5000 VhT. Maximum intensity was kept at 50 µA/strip. Before the second dimension, the strips were incubated 15 min in reduction buffer (130 mM DTT, 6 M urea, 0.373 M Tris-HCl pH 8.8, 20% glycerol, 2% SDS) and 15 min in an alkylation buffer (135 mM iodoacetamide, 6 M urea, 0.373 M Tris-HCl pH 8.8, 20% glycerol, 2% SDS). For the second dimension separation, IPG strips were loaded on NuPAGETM 4–12% Bis-Tris ZOOMTM Gel (Invitrogen) and the gels were run in the MES SDS Running Buffer (Invitrogen) at 200 V until Bromophenol Blue reached the front. The gels were stained with Coomassie Brilliant Blue.

Protein spots of interest were excised from the gel. Gel plugs were washed 3 times under agitation (600 RPM) with first 50 µL of 50 mM NH_4_HCO_3_ and then 50 µL of 50 mM NH_4_HCO_3_/ACN (Biosolve, ULC MS grade) 50% (*v*/*v*), cysteine residues were reduced with a 10 mM DTT solution for 40 min at 56 °C followed by alkylation with 55 mM iodoacetamide at RT in the dark. Digestion was performed overnight with 3 µL of 10 ng/μL of chymotrypsin (Thermo Scientific) in 25 mM NH_4_HCO_3_, 5 mM CaCl_2_ pH 8 buffer. The resulting peptides were extracted with 20 µL of aqueous solution at 1% TFA. 9 µL were injected on the previously described LC/MS system with a total LC run of 60 min of following linear gradient: initial conditions 98% A, 5 min 93% A, 30 min 60% A and 33 min 15% A. The mass spectrometer method is a TopN-MSMS method where *n* was set to 10.

### 2.6. Functional Assays

Tissue culture plastic (TCP) surfaces were coated by 2 h incubation at 37 °C with 100 μL/cm^2^ of rat tail collagen I (Gibco) or solubilized HL-ECM, both used at the optimal concentration of 100 μg/mL (Figure S1C) as determined by protein dosage using the Micro BCA protein assay kit (Thermo Scientific) according to the manufacturer’s instructions. Coated plates were thoroughly washed with PBS and stored at 4 °C overnight. For medium supplementation, 1 mg/mL solubilized HL-ECM was diluted 1:10 in culture medium to reach a final concentration of 100 μg/mL.

Thawed HEPs were seeded at a density of 66,666 viable cells/cm^2^ on coated TCP in cryopreserved hepatocyte plating medium (Gibco), replaced 6 h after seeding by glutamine-free William’s E medium (Gibco) supplemented with 10% FBS (Gibco), 1% penicillin-streptomycin solution (Gibco), insulin 0.016 U/mL (Eli Lilly, Indianapolis, IN, USA) and 0.05% dexamethasone (Rotexmedica, Hamburg, Germany). Plating efficiency was determined by dosing DNA with the CyQUANT cell proliferation assay kit according to the manufacturer’s instructions 24 h and 5 days after seeding. The viability at day 1 and 5 was measured by resazurin reduction using the PrestoBlue cell viability reagent (Invitrogen) according to the manufacturer’s instructions (24 h incubation, λex = 560 nm and λab = 620 nm). Urea was quantified in snap-frozen supernatants collected after 5 days of culture using the QuantiChrom urea assay kit (BioAssay Systems, Hayward, CA, USA) according to the manufacturer’s instructions. The results were normalized to the number of cells as assessed by DNA dosage. All colorimetric and fluorimetric measurements were performed on a Victor X4 spectrophotometer (PerkinElmer, Waltham, MA, USA).

HSCs were used in experiments after at least two passages post-isolation. They were seeded at a density of 10,000 viable cells/cm^2^ on non-coated TCP (unless stated otherwise) in Dulbecco’s modified Eagle medium (DMEM), 4.5 g/L glucose, 25 mM HEPES (Lonza) supplemented with 10% FBS, 1% UltraGlutamine solution (Lonza), 1% penicillin-streptomycin solution and where indicated, with 100 µg/mL HL-ECM solution. The metabolic activity of the cells was evaluated 1, 3 and 7 days after seeding by resaruzin reduction (2 h incubation, fluorescence detection).

CD146^+^ (LS)ECs isolated by MACS from cryopreserved NPF were maintained in culture on coated TCP for up to 3 days in supplemented endothelial growth medium 2 (PromoCell, Heidelberg, Germany) at a density of 50,000 total cells/cm^2^.

CD146^−^CD14^+^ MPs isolated by MACS from cryopreserved NPF were seeded at a density of 50,000 total cells/cm^2^ on uncoated TCP in DMEM, 4.5 g/L glucose, 2 mM glutamine (Gibco) supplemented with 10% FBS and 1% penicillin-streptomycin solution. Cells were washed and pictures were taken 24 h after seeding.

### 2.7. RNA Extraction, Reverse Transcription and RT-qPCR

RNA was extracted from freshly isolated or cultured cells at indicated time points using the TriPure isolation reagent (Roche, Basel, Switzerland) according to the manufacturer’s instructions. The remaining genomic DNA was digested by DNase I (Invitrogen). RNA was retrotranscribed using the high-capacity cDNA reverse transcription kit (Applied Biosystems, Foster City, CA, USA), and RT-qPCR was performed using TaqMan universal MasterMix (Applied Biosystems) and TaqMan probes listed in Table S2 according to the manufacturer’s instruction, on a StepOnePlus real-time PCR machine (Applied Biosystems). Relative gene expression was determined using the ΔΔCt method using *POP4* and *PPIA* as housekeeping genes.

### 2.8. Statistical Analysis

Unless stated otherwise, results were obtained from independent experiments performed on at least 3 different donors. Graphs show the mean ± SEM. Statistical analysis was performed with GraphPad Prism v5. A Student’s *t*-test, one-way ANOVA with a Dunnett’s post-hoc test or two-way ANOVA with Bonferroni’s post-hoc test were applied when appropriate.

## 3. Results

### 3.1. Enrichment of Human Liver Cells from Cryopreserved PF and NPF

To address the lack of adequate cryopreservation conditions for human liver cells, we tested different media for their ability to cryopreserve the PF and the NPF obtained directly after liver digestion and low-speed centrifugal separation. This comparative analysis demonstrated the suitability of a medium standardly used in our biobank for the cryopreservation of PF, consisting of 75% IGL-1, 15% Hibumine and 10% DMSO, for the cryopreservation of the heterogeneous NPF. Indeed, cryopreservation with this medium had virtually no impact on the cell viability (>95% viable cells, Table S3). To assess whether the different major liver cell populations can be isolated from the cryopreserved liver cell fractions, we adapted a protocol previously applied to fresh human liver material [22] and identified the purified cell populations by a comparative analysis of the expression of cell-type specific genes by qPCR: albumin (*ALB*), cytochrome P450 3A4 (*CYP3A4*), hepatocyte nuclear factor 4α (*HNF4A*) and glucose-6-phosphatase catalytic subunit (*G6PC*) for HEPs, cytoglobin (*CYGB*) for HSCs, NO synthase 3 (*NOS3*) as a pan EC marker, stabilin 1/2 (*STAB1/2*), Fcγ receptor 2b (*FCGR2B*) and lymphatic vessel endothelial hyaluronan receptor 1 (*LYVE1*) for LSECs and *CD163* for MPs [14,16,22,27,28].

**Figure 1:**
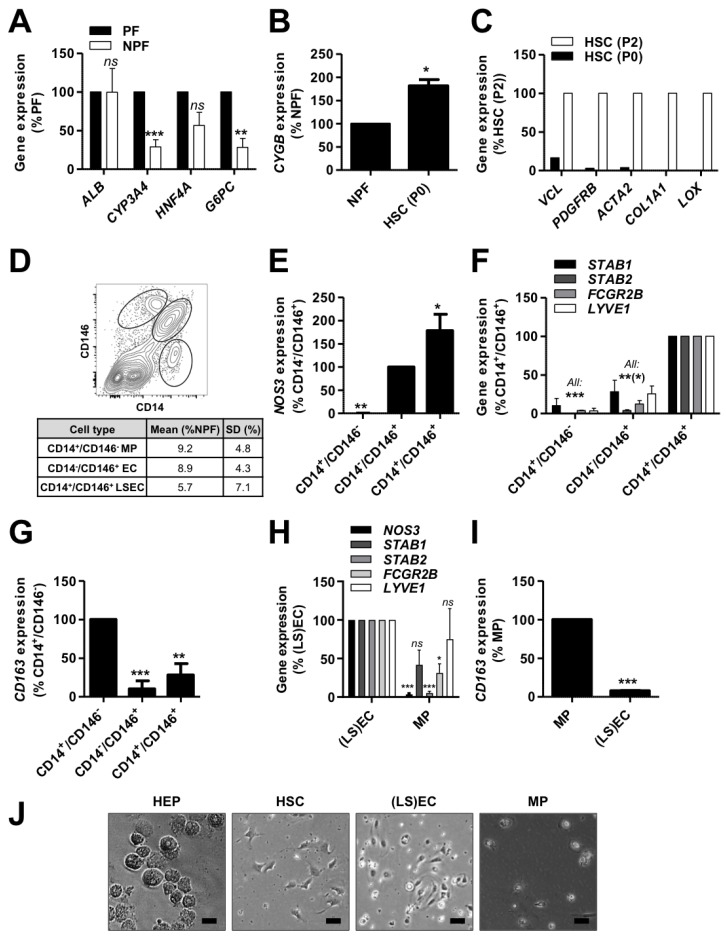
Isolation of human primary liver cells. Human primary liver cells were isolated from cryopreserved material of 4 donors (155, 156, 164 and 173). Gene expression was evaluated by qPCR (**A**) in hepatocytes (HEPs, *n* = 4), (**B**) in hepatic stellate cells (HSCs) just after isolation (P0, *n* = 3) and (**C**) after 2 passages on tissue culture plastic (P2, *n* = 2) for representative markers. (**D**) Representative flow cytometry dot plot (upper panel) and average yields after non-parenchymal fraction (NPF) FACS according to CD146 and CD14 expression. (**E**–**G**) Gene expression was evaluated by qPCR in CD14^+^CD146^−^ macrophages (MPs), CD14^−^CD146^+^ endothelial cells (ECs), CD14^+^CD146^+^ liver sinusoidal ECs (LSECs) and CD14^−^CD146^−^ other cells for representative markers (*n* = 3). (**H**,**I**) After magnetic beads-activated cell sorting (MACS) for (i) CD146 and (ii) CD14, gene expression was assessed by qPCR in CD146^+^ (LS)ECs and CD146^−^CD14^+^ MPs for representative markers (*n* = 3−4). (**J**) Representative pictures of freshly isolated cells (scale bar = 50 μm). All: results were analyzed per donor and are expressed as percentage of expression in the indicated fraction (expected maximal expression), graphs show mean ± SEM, Student’s *t*-test (**A**,**B** and **H**,**I**) or one-way ANOVA with Dunnett’s post-hoc test (**E**–**G**), ns *p* > 0.05, * *p* < 0.05, ** *p* < 0.01, *** *p* < 0.005. *ACTA2*: actin α2, *ALB*: albumin, *COL1A1*: collagen type 1 α1 chain, *CYGB*: cytoglobin, *CYP3A4*: cytochrome P450 3A4, *FCGR2B*: Fcγ receptor 2b, *G6PC*: glucose 6 phosphatase catalytic subunit, *HNF4A*: hepatocyte nuclear factor 4 α, *LOX*: lysyl oxidase, *LYVE1*: lymphatic vessel endothelial hyaluronan receptor 1, *NOS3*: NO synthase 3, *PDGFRB*: PDGF receptor β, *STAB*: stabilin, *VCL*: vinculin.

The NPF showed lower *CYP3A4*, *HNF4A* and *G6PC* expression levels in comparison with the PF (Figure 1A), confirming the relative depletion and enrichment of HEPs in the supernatant and pellet, respectively, following low speed centrifugation of the total liver cell suspension before cryopreservation. The expression of *ALB* was however found to be similar in both fractions (Figure 1A). Given the high expression level of *ALB* in HEPs and the relative abundance of HEPs to the other liver cell types, this result indicates that the low speed centrifugation steps do not fully deplete the NPF from HEPs. This is in accordance with our microscopic observations.

The centrifugation of the NPF on a density gradient to enrich for lipid-droplet loaded quiescent HSCs resulted in the enrichment of *CYGB*-expressing cells, a marker of HSCs (Figure 1B) [29]. Upon culture on TCP, the HSCs underwent spontaneous activation, i.e., they acquired a fibrogenic, myofibroblast-like phenotype and expressed high levels of vinculin (*VCL*), PDGF receptor β (*PDGFRB*), actin α2 (*ACTA2*), collagen I α1 chain (*COL1A1*) and lysyl oxidase (*LOX*) (Figure 1C). After two passages (HSC P2), the cells formed a phenotypically homogeneous cell population.

Whereas the purification of MPs and (LS)ECs from fresh NPF has been proposed by sequential sorting of respectively CD14 and CD146 positive cells by MACS [22], a recent study shows that a subset of LSECs also expresses CD14 [30]. We conducted a FACS analysis of freshly thawed NPF for the markers CD14 and CD146 (Figure 1D) and subsequently sorted the cells for gene expression analysis (Figure 1E–G). We indeed found a CD14^+^CD146^+^ cell population, specifically expressing the pan-endothelial marker *NOS3* and the LSEC-markers *STAB1*, *STAB2*, *FCGR2B* and *LYVE1*. In contrast, the CD14^−^CD146^+^ population expressed *NOS3* but no LSEC or MP markers, while the CD14^+^CD146^−^ population only expressed high levels of the MP-specific marker *CD163*. Based on these findings, we showed that sequential MACS from cryopreserved NPF according to (i) CD146 and (ii) CD14 expression can efficiently separate (LS)ECs from MPs (Figure 1H,I). While HSCs are reported to also express *CD146* [30], our results show no enrichment in *CYGB* expressing cells in the CD146^+^ (LS)EC fraction (Figure S2). This could at least in part be explained by our tissue dissociation protocol. The optimal isolation of HSCs indeed requires a pronase digestion step [31,32]. However, as pronase destroys parenchymal cells, it is not compatible with the isolation of hepatocytes. The absence of pronase in our digestion protocol probably negatively affects the yield of HSCs in the resulting total cell suspension, potentially explaining the low degree of HSC contamination in the CD146^+^ fraction.

While the MACS procedure based on CD14 and CD146 does not discriminate between ECs and LSECs, we observed a better throughput and post-isolation recovery of the cells than with the FACS-based procedure. The MACS procedure is thus more suitable for downstream applications. Table S4 displays the respective yields for each cell type while Figure 1J shows representative pictures of the different isolated cell types using centrifugation and MACS: polygonal mono- or binucleated HEPs, mesenchymal HSCs, elongated and dense (LS)ECs and vacuolar MPs with a clear nucleus.

### 3.2. Proteomic and Peptidomic Characterization of Solubilized HL-ECM

**Figure 2:**
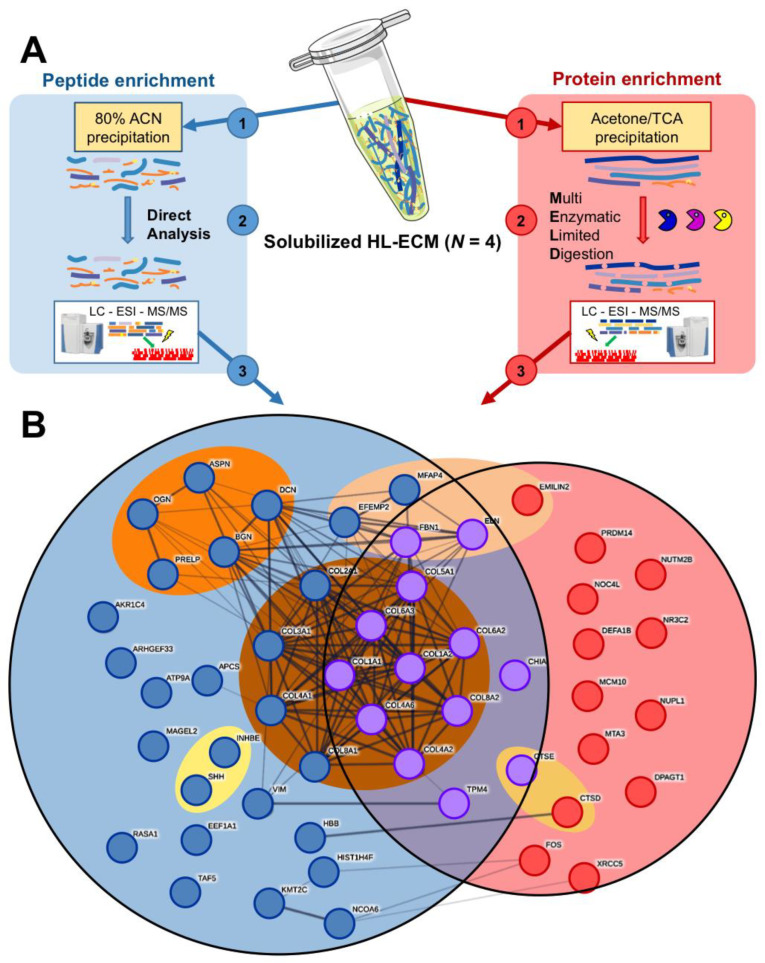
Composition of solubilized human liver extracellular matrix (HL-ECM). A proteomic analysis was performed on solubilized human liver extracellular matrix (HL-ECM), derived from 4 donors (154, 155, 158 and 159). (**A**) Schematic representation of the methodology applied to separately analyze peptide- and protein-enriched fractions. (**B**) String plot of the 52 proteins identified by mass spectrometry in the solubilized HL-ECM: each circle represents 1 protein; each line represents 1 identified protein–protein interactions in the String database; blue, red and purple filling indicate whether the protein was identified in the peptide, protein or both fractions; proteins were grouped in sets proposed in the Matrisome project. ACN: acetonitrile, ESI: electrospray ionization, LC: liquid chromatography, MS: mass spectrometry, TCA: trichloroacetic acid.

Cryopreserved liver fragments from four donors were decellularized and solubilized to be used as a surface coating for the culture of human primary liver cells. The applied decellularization protocol (Figure S1A) allowed for complete decellularization, as quantification for double-strand DNA was found in each preparation to be below the critical threshold of 50 ng/mg ECM [23,33], within a range of 0.9 ± 0.0 and 19.6 ± 0.5 ng/mg ECM. Yields are shown in Table S5. The lyophilized and solubilized HL-ECM showed abundant collagen content (Figure S1B) and was further characterized by proteomics. We conducted a distinct qualitative analysis per donor of both the peptide fraction (below 10 kDa) and the protein fraction (above 10 kDa, Figure 2A). In solubilized

HL-ECM we identified 52 proteins in the whole pool of donors: 26 (50%) were exclusively present in the peptide fraction, 13 (25%) were exclusively in the protein fraction and 13 (25%) were shared by both fractions (Figure 2B, Table 1). Just over 60% (*n* = 32) of the proteins identified in the solubilized HL-ECM were membrane and extracellular proteins. In lyophilized HL-ECM, we identified 267 proteins in the whole pool of donors (Table S6): 224 exclusively in the peptide fraction (84%), 10 exclusively in the protein fraction (4%) and 33 in both fractions (12%). Membrane and extracellular proteins represented 25% (*n* = 68) of all the detected proteins. A comparative analysis between lyophilized and solubilized HL-ECM revealed that (i) most extracellular components identified in solubilized HL-ECM were also detected in the lyophilized ECM (24 shared extracellular components out of 32 in solubilized HL-ECM), (ii) lyophilized HL-ECM displayed a wider diversity in extracellular components than solubilized HL-ECM (68 versus 32 extracellular components) and (iii) several extracellular components detected in the protein fraction of the lyophilized HL-ECM were only detected in the peptide fraction of the solubilized HL-ECM (including the proteoglycan mimecan (OGN) and four collagen chains). Together, our observations suggest that HL-ECM digestion with pepsin, the most widely used enzyme to solubilize tissue-derived ECM [34], could at least partially decrease component diversity and integrity in solubilized HL-ECM. However, 1D SDS-PAGE showed the presence of protein bands above 62 kDa, thereby confirming the resistance of some proteins to decellularization and digestion with pepsin (Figure S3A).

HL-ECM components were classified according to the items proposed by the Matrisome Project [35,36] (Figure 2B, Table 1). Among them, a large cluster of “collagens” (12 collagen types—23% total identified proteins—present in both fractions) appeared in solubilized HL-ECM, where the collagens type I α1 (COL1A1—45.4% of the quantifiable protein mass in the protein fraction) and α2 (COL1A2—54.3% of the quantifiable protein mass in the protein fraction) chains were the most abundant. Importantly, only COL1A1, COL1A2 and FBN1 were identified by three unique peptides, and thus quantifiable, in the protein fraction. COL1A1 and COL1A2 were also the only two spots identified in a 2D SDS-PAGE of the solubilized HL-ECM, thereby confirming their relative abundance and their resistance to decellularization and pepsin digestion (Figure S3B). In contrast, “proteoglycans”—asporin (ASPN), biglycan (BGN), decorin (DCN), osteoglycin (OGN) and prolargin (PRELP)—and “secreted factors”—inhibin β E (INHBE) and Sonic hedgehog (SHH)—were only found in the peptide fraction, suggesting a loss of the integrity of those proteins. Additionally, HL-ECM solution featured “ECM glycoproteins”, notably elastin (ELN—present in both fractions), “ECM regulators” and intracellular proteins (20 proteins—38% total identified proteins).

We found by dosing the protein content retained on TCP following coating with different concentrations of solubilized HL-ECM that a plateau in protein adsorption started at 50 µg/mL (Figure S1C). We conducted all subsequent experiments on plates coated with solubilized HL-ECM at the highest tested concentration, i.e., 100 µg/mL, to ensure maximal protein adsorption on the plates.

### 3.3. Evaluation of Solubilized HL-ECM for Primary Human Liver Cell Culture

**Figure 3:**
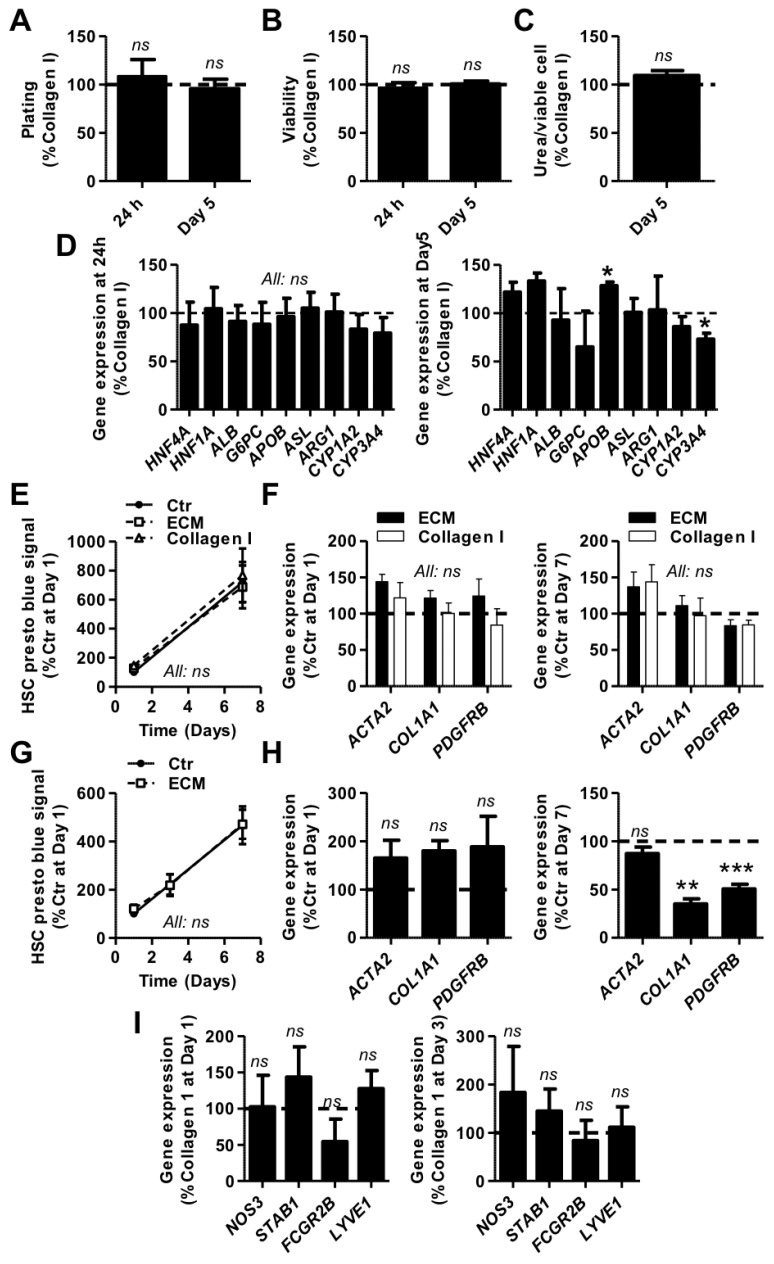
Human liver cell culture on a solubilized HL-ECM coating. (**A**–**D**) Hepatocytes (HEPs) from 3 donors (32, 155 and 158) were seeded on rat tail collagen I (Collagen I)- and solubilized human liver extracellular matrix (ECM)-coated tissue culture plastic and maintained in culture for up to 5 days. (**A**) Plating was evaluated after 24 h and 5 days of culture by DNA dosage. (**B**) Viability was evaluated after 24 h and 5 days of culture by resazurin reduction. (**C**) Urea was measured in culture supernatant after 5 days of incubation and normalized to viable HEP number. (**D**) Expression of key markers of hepatocyte functions was assessed by qPCR after 24 h and 5 days of culture (right panel: only statistically significant differences are indicated). (**E**,**F**) HSCs from 3 donors (94, 97 and 98) were grown on tissue culture plastic surface (i) uncoated (Ctr) or (ii) coated with solubilized human liver ECM or (iii) with a rat tail collagen I solution. (**E**) The number of viable HSCs over time was evaluated by resazurin reduction. (**F**) Gene expression was evaluated after 24 h and 7 days of culture by qPCR for markers of activated HSCs. (**G**,**H**) HSCs from 3 donors (94, 97 and 98) were grown on a tissue culture plastic surface in culture medium (i) non-supplemented (Ctr) or (ii) supplemented with 100 μg/mL solubilized human liver ECM. (**G**) The number of viable HSCs over time was evaluated by resazurin reduction. (**H**) Gene expression was evaluated after 24 h and 7 days of culture by qPCR for markers of activated HSCs. (**I**) (LS)ECs from 3 donors (156, 164 and 173) were seeded at the same density on rat tail collagen I- and solubilized human liver ECM-coated tissue culture plastic and maintained in culture for up to 3 days. Gene expression was evaluated after 24 h and 3 days of culture by qPCR for EC and LSEC markers. All: *n* = 3, results were analyzed per donor, graphs show mean ± SEM, results are expressed as % Collagen I or % Ctr, Student’s *t*-test (All except **E** and **G**) or 2-way ANOVA with Bonferroni’s post-hoc test (**E** and **G**) versus Collagen I or Ctr value, ns *p* > 0.05, * *p* < 0.05, ** *p* < 0.01, *** *p* < 0.005. *ACTA2*: actin α2, *ALB*: albumin, *APOB*: apolipoprotein B, *ARG1*: arginase 1, *ASL*: argininosuccinate lyase, *COL1A1*: collagen type 1 α1 chain, *CYP*: cytochrome P450, *FCGR2B*: Fcγ receptor 2b, *G6PC*: glucose-6-phosphatase catalytic subunit, *HNF4A*: hepatocyte nuclear factor 4α, *HNF1A*: hepatocyte nuclear factor 1α, *LYVE1*: lymphatic vessel endothelial hyaluronan receptor 1, *NOS3*: NO synthase 3, *PDGFRB*: PDGF receptor β, *STAB*: stabilin.

Human primary HEPs are known to lose their morphology and metabolic functions within the first couple of days of in vitro culture [8]. Recent studies suggest that animal-derived liver ECM, when used as a coating or media supplement in 2D cultures, can favorably affect the production of albumin and urea in rat and porcine primary hepatocytes [16,17,18]. We sought to investigate whether a HL-ECM coating could similarly affect the functionality of thawed human primary HEPs and represent a better alternative than the traditionally used rat tail collagen I coating. Our results showed that, in comparison with the rat tail collagen I coating, the HL-ECM coating did not positively nor negatively influence the adhesion (Figure 3A) and viability (Figure 3B) of human HEPs up to 5 days of culture. After 5 days of culture, the amount of urea in the supernatant of both conditions was similar (Figure 3C). Finally, we did not observe any major difference in the expression of a panel of genes representative of HEP specific functions, including genes coding for specific transcription factors (hepatocyte nuclear factor 1A and 4A—*HNF1A* and *HNF4A*), plasma proteins (*ALB*) and enzymes involved in gluconeogenesis (glucose-6-phosphatase catalytic subunit—*G6PC*), lipid metabolism (apolipoprotein B—*APOB*), the urea cycle (argininosuccinate lyase and arginase 1—*ASL* and *ARG1*) and drug metabolism (cytochrome P450 1A2 and 3A4—*CYP1A2* and *CYP3A4*, Figure 3D). Taken together, our results show identical properties for HEPs cultured on HL-ECM and rat tail collagen I coatings.

The phenotypic changes that human primary HSCs undergo rapidly upon a culture on hard substrates hampers the study of their physiologic state [37]. Given recent reports showing that ECM components can negatively regulate the activation of rat HSCs and maintain their quiescence-associated phenotype [38,39], we investigated the effects of both coating and culture medium supplementation with solubilized HL-ECM on the activation state of primary human HSCs for up to 7 days of culture on uncoated TCP. We found that neither HL-ECM nor rat tail collagen I coating had influence on HSC parameters as compared to TCP (Figure 3E,F). In contrast, whereas medium supplementation with HL-ECM had no influence on HSC proliferation (Figure 3G) and *ACTA2* gene expression, we observed a decrease of *COL1A1* and *PDGFRB* gene expression after 7 days of culture (Figure 3H), indicative of a less activated and fibrogenic cell state.

Several approaches have been considered in order to maintain LSEC phenotypic features in culture [40], among which the use of ECM and basal lamina components [15]. In this particular context, we hypothesized that the culture of human primary (LS)ECs on a HL-ECM coating could improve the maintenance of their specific characteristics in comparison with a classical rat tail collagen I coating. To do so, we evaluated the expression of genes representative of the differentiated LSEC phenotype (i.e., scavenger receptors (*STAB1*, *STAB2* and *FCGR2B*), nitric oxide synthase (*NOS3*) and the hyaluronan receptor *LYVE1*) immediately after isolation and up to 3 days in culture by qPCR (Figure 3I). Our results showed no differences in gene expression patterns between both coatings.

## 4. Discussion

In the present study, we validated an original and efficient method to simultaneously isolate the major human primary liver cell populations from cryopreserved total liver cell suspensions, and we produced solubilized HL-ECM and characterized its peptidomic and proteomic composition prior to evaluating its potential for the culture of human liver cells.

Whereas protocols for HEP cryopreservation have been extensively described in the literature [6], conditions that allow for the efficient preservation of the NPF have barely been studied. To the best of our knowledge, we for the first time describe a condition, i.e., a combination of cell density, cryopreservation medium and cooling procedure, that is suited for the preservation of both the PF and the NPF, thereby alleviating the dependence on the availability of fresh material for the simultaneous isolation of parenchymal and non-parenchymal liver cells for research and/or clinical applications. HEPs and HSCs isolated from the cryopreserved liver cell suspensions using previously described procedures [10,22,41,42] showed expression of characteristic markers and displayed typical morphology and metabolic features. We further adapted an existing protocol for the isolation of (LS)ECs and MPs [22] and applied it for the purification of cells from the cryopreserved material. The existence of a CD14^+^ LSEC population evidenced by immunostaining of human liver sections [30] was confirmed by our FACS analysis and drove the modifications in our purification procedure. While the FACS-based procedure yielded cell preparations of excellent purity and the possibility to discriminate LSECs from macro-vascular ECs, it negatively affected the viability of the cells and returned limited cell yields. In contrast, the (LS)EC fraction composed of both LSECs and macro-vascular ECs and the MP fraction purified by MACS displayed a good recovery in culture and was thus considered for further applications. While our results show that our procedure allowed for the isolation of the main liver cell populations from heterogeneous and cryopreserved liver cell suspensions, the yields obtained in our experiments were significantly lower than those described by Werner et al. on fresh material [22]. As we had not carried out cell purification experiments on fresh material from the same donors, it was not clear whether this difference was caused by the cryopreservation itself or if it was merely due to upstream differences in the tissue sampling and tissue dissociation protocol.

Using the described cell preservation and purification protocol, we designed experiments to provide a first series of results on the influence of HL-ECM on the phenotype of human primary liver cells. For this purpose, we successfully isolated HL-ECM and produced solubilized HL-ECM that can be easily stored, transferred and used as a coating or cell culture media supplement. It is important to note that we were chiefly interested in assessing the impact of HL-ECM in a conformation that would not limit further downstream applications of the cells for research or clinical applications. Although several studies have shown the value of complex in vitro culture techniques such as spheroid or dynamic cultures to maintain primary liver cell functions [43,44], they do not readily lend themselves to larger scale applications. Similarly, while culturing the cells in a 3D conformation in HL-ECM based gels or scaffolds arguably has more potential to positively affect the phenotype and functionality of the cells, these methods significantly limit downstream applications and were thus not considered in the scope of our study.

In view of providing a first in depth molecular characterization of the HL-ECM, we implemented an original method of separation of peptides and proteins followed by a mass spectrometry analysis. In addition to many intracellular proteins (as reported by other studies in animal liver and human organs [18,36]), we globally identified similar extracellular components than those reported by The Matrisome Project [35,36], in which total human, non-decellularized liver protein extracts depleted in intracellular proteins by centrifugation steps were analyzed. Notable differences in our study include the non-detection of “ECM-affiliated proteins” and “secreted factors” probably lost during organ decellularization, and the detection of few additional ECM components such as the LAMA5 glycoprotein. Our differential analysis of peptides and proteins allows us to understand the impact of the decellularization protocol and pepsin digestion on protein integrity. For example, most ECM proteoglycans were only detected in the peptide fraction, thereby suggesting that these proteins are more sensitive to decellularization than collagens. After digestion with pepsin, the diversity and integrity of ECM components decreased, resulting in a mixture of various proteins among which abundant collagen proteins and peptides.

We found that while the HL-ECM coating was adequate to support the adhesion and viability of the different tested cell types, it did not improve the viability nor did it induce any pronounced positive phenotypical changes in the cells compared to cells grown under control conditions. To our knowledge, our study is the first to provide experimental results using ECM and cells exclusively derived from human livers. We have however worked at concentrations for TCP coating and medium supplementation that are similar to those reported in previous studies using animal cells to allow comparison of our results [16,17]. While some of those studies suggested potential benefits to the use of liver-derived ECM compared to other traditional coatings for the culture of HEPs, those benefits were limited in amplitude, species- or time-dependent, and associated with high variability. We therefore consider our results to be largely in line with previous reports and conclude that while HL-ECM is a suitable substrate to culture human HEPs, we found that it was not significantly better than the widely used rat tail collagen I coating. However, for the (LS)ECs, our observations differ from another study where primary rat LSECs grown on a rat liver ECM-derived substrate maintained their specific marker expression and fenestrations for at least 3 days of culture, as compared to collagen I [15]. The composition of the solubilized HL-ECM might at least in part explain the limited impact observed in our experiments; our results indicate a loss or degradation of some potentially interesting ECM components following pepsin digestion. While peptides and proteins reported in the lyophilized HL-ECM but not detected in the solubilized HL-ECM could still be present as traces, their amount might not be sufficient to induce a biologic effect. Of note, we also observed important differences in HL-ECM composition (and consequently in derived solutions) between donors, in accordance with previous results [45], attributed to the heterogeneity of liver donors and inherent technical variations during decellularization. As for the composition of the ECM, variability in gene expression between different cell preparations is likely due to intrinsic differences between donors and inherent variations in the initial steps of processing the human livers. Similarly to HEPs and LSECs, the HL-ECM coating did not downregulate the proliferation rate and fibrogenic gene expression profile of activated HSCs [38]. However, when solubilized HL-ECM was supplemented in the culture medium of non-coated TCP cultured HSCs, as previously described for primary rat liver cells [17], we observed a decrease in the expression of *PDGFRB* and *COL1A1*, respectively coding for one of the main fibrogenic triggers and products, suggesting a partial resolution of the fibrogenic phenotype [10,46]. In contrast, we detected no impact on the expression of *ACTA2*, coding a component of the contractile machinery, indicating the maintenance of the myofibroblastic phenotype. Of note, *PDGFRB* downregulation was not associated with a decrease of HSC proliferation, a functional hallmark of activated HSCs [47]. A potential explanation for the observed difference in effect between coated and medium-supplemented HL-ECM is that medium supplementation allowed us to expose the cells to a higher dose of solubilized ECM components. Of note, the concentrations of HL-ECM used for TCP coating resulted in an adsorption plateau at 1 μg/cm^2^, i.e., a lower dose than as a medium supplement. Altogether, we think that the absence of relevant biological effect of solubilized HL-ECM is linked to an insufficient dose of active ECM components, which itself depends on the liver donor, the solubilization process and the application to liver cell culture.

Importantly, our study highlights the need for softer decellularization and solubilization methods. A limited number of studies used physical methods such as homogenization prior to the decellularization process (enzymatic digestion and/ or detergents) in order to increase the contact surface, remove the lipids and improve the penetration of solutions while minimizing the exposure time to detergents and maintaining the microstructure of the tissue [48,49]. Alternatively, the use of supercritical carbon dioxide has been suggested for the delipidation and decellularization of tissues, in order to reduce the duration of the process and to avoid the use of harsh chemicals [50,51]. For ECM solubilization, whereas pepsin/HCl is the gold standard, we clearly show that this approach preserves the collagens but is destructive for other potentially bioactive ECM components. While different other enzymes have been proposed for the removal of cellular material, including chymotrypsin, dispase and phospholipase A2 [52,53,54], it remains to be demonstrated that these are better suited alternatives.

Despite the translational potential and the importance of decellularized liver ECM in the biomedical research and tissue engineering fields, there are still many challenges to overcome regarding the limited access to human liver fragments, the inter-donor variability of composition and the technical difficulties related to tissue decellularization and solubilization. We strongly believe that exploiting the best of HL-ECM properties requires the identification of more efficient procedures for the preservation of its bioactive components.

## Figures and Tables

**Table 1:** Composition of solubilized HL-ECM. Summary of the proteomic analysis of solubilized HL-ECM: gene and protein name, classification according to the Matrisome project, fraction where the protein was identified.

Gene	Protein	Division	Function	Fraction
*COL1A1*	Collagen I α1 chain	Core matrisome	Collagen	Both
*COL1A2*	Collagen I α2 chain			
*COL2A1*	Collagen II α1 chain			
*COL3A1*	Collagen III α1 chain			
*COL4A2*	Collagen IV α2 chain			
*COL4A6*	Collagen IV α6 chain			
*COL5A1*	Collagen V α1 chain			
*COL6A3*	Collagen VI α3 chain			
*COL4A1*	Collagen IV α1 chain			Peptide
*COL6A2*	Collagen VI α2 chain			
*COL8A1*	Collagen VIII α1 chain			
*COL8A2*	Collagen VIII α2 chain			
*EMILIN2*	EMILIN 2	Core matrisome	ECM glycoprotein	Protein
*ELN*	Elastin			Both
*FBN1*	Fibrillin 1			
*EFEMP2*	EGF-containing fibulin-like ECM protein			Peptide
*MFAP4*	Microfibril-associated glycoprotein 4			
*ASPN*	Asporin	Core matrisome	Proteoglycan	Peptide
*BGN*	Biglycan			
*DCN*	Decorin			
*OGN*	Mimecan			
*PRELP*	Prolargin			
*VTN*	Vitronectin			
*CTSD*	Cathepsin D	Matrisome-associated	ECM regulator	Protein
*CTSE*	Cathepsin E			Both
*SHH*	Sonic hedgehog	Matrisome-associated	Secreted factor	Peptide
*INHBE*	Inhibin β E			
*NR3C2*	Mineralocorticoid receptor	Matrisome-unrelated	Membrane receptor	Protein
*DEFA1*	Neutrophil defensin 1	Matrisome-unrelated	Secreted protein	Protein
*CHIA*	Acidic mammalian chitinase			Both
*APCS*	Serum amyloid P-component			Peptide
*HBB*	Hemoglobin subunit β			
*DPAGT1*	DPAG phosphotransferase	Intracellular		Protein
*FOS*	Proto-oncogene c-Fos			
*MCM10*	MCM 10			
*MTA3*	Metastasis-associated protein 3			
*NOC4L*	Nucleolar complex protein 4			
*NUP58*	Nucleoporin p58/p45			
*NUTM2B*	NUT family member 2B			
*PRDM14*	PR domain zinc finger protein 14			
*XRCC5*	X-ray repair cross-complementing protein 5			
*TPM4*	Tropomyosin α4 chain			Both
*ARHGEF33*	Rho guanine nucleotide exchange factor 33			Peptide
*ATP9A*	Probable phospholipid-transporting ATPase IIA			
*EEF1A*	Elongation factor 1α			
*KMT2C*	Histone-lysine N-methyltransferase 2C			
*HIST1H4*	Histone H4			
*MAGEL2*	MAGE-like protein 2			
*NCOA6*	Nuclear receptor coactivator 6			
*RASA1*	Ras GTPase-activating protein 1			
*TAF5*	Transcription initiation factor TFIID subunit 5			
*VIM*	Vimentin			
*KRT1*	Keratin type II cytoskeletal 1	Probable contaminant		Protein
*KRT9*	Keratin type I cytoskeletal 9			
*KRT10*	Keratin type I cytoskeletal 10			
*ALB (B. taurus)*	Serum albumin	Contaminant		Both
*PGA4*	Pepsin A4			
*DNASE1*	Deoxyribonuclease 1			Peptide

## Supplementary Materials

The following are available online at https://www.mdpi.com/2073-4409/9/6/1357/s1, Figure S1: Human liver decellularization and HL-ECM solubilization, Figure S2: Expression of the HSC-specific marker *CYGB* in the MACS-sorted CD146^+^ cell fraction, Figure S3: Electrophoresis on HL-ECM, Table S1: Donor list, Table S2: Taqman assay list, Table S3: Viability of NPF cell suspensions before and after cryopreservation in different media, Table S4: Yields of human primary liver cells, Table S5: Yields of lyophilized HL-ECM, Table S6: Extended proteomic data.
