# Supplementary material for: Human Liver-Derived Extracellular Matrix for the Culture of Distinct Human Primary Liver Cells
Source: Cells. 2020 May 30;9(6):1357. doi: 10.3390/cells9061357 (PMC7349413; doi:10.3390/cells9061357)
Supplement: Supplementary file 1 [file cells-09-01357-s001.zip › Legend of supplementary Figures and Tables_revised.docx]

**Legend of the supplementary figures and tables.**

**Figure S1 – Human liver decellularization and HL-ECM solubilization. (A)** Pictures of the workflow for human liver decellularization and solubilized human liver extracellular matrix (HL-ECM) production. **(B)** Representative Sirius Red staining of cryosections of lyophilized (left panel) and solubilized (right panel) HL-ECM (scale bar: 50 μm). **(C)** Protein adsorbed on tissue culture plastic after 2h incubation with solubilized human liver ECM at various concentrations.

**Figure S2 - Expression of the HSC-specific marker *CYGB* in the MACS-sorted CD146+ cell fraction.** Human primary liver cells were isolated from cryopreserved material of 3 donors (155, 156 and 173). The expression of the hepatic stellate cell (HSC) marker cytoglobin (*CYGB*) was assessed by qPCR in CD146+ (liver sinusoidal) endothelial cells ((LS)ECs) isolated by MACS (N = 3) and compared to the parental non-parenchymal fraction (NPF). Results were analyzed per donor and are expressed as percentage of expression in corresponding HSCs immediately after isolation (P0, expected maximal expression), graph shows mean ± SEM, Student’s t-test, *ns* p > 0.05.

**Figure S3 – Electrophoresis of HL-ECM. (A)** Representative 1D SDS-PAGE of proteins in lyophilized human liver extracellular matrix (HL-ECM) and corresponding solubilized HL-ECM revealed by Coomassie blue staining. **(B)** Representative 2D SDS-PAGE of proteins in lyophilized HL-ECM and corresponding solubilized HL-ECM revealed by Coomassie blue staining. The 2 spots identified in the latter were further identified by mass spectrometry (MS) as collagen 1 α1 (COL1A1) and α2 (COL1A2) chains. MW: molecular weight ladder.

**Table S1 – Donor list.** Age, sex and liver condition of human livers used in this study for cell and extracellular matrix isolation. F: female, M: male, OTC: ornithine carbamoyltransferase, MSUD: maple syrup urine disease.

**Table S2 – TaqMan assay list.** Gene abreviation, gene name, cell type where maximal expression is expected, gene function, and TaqMan assay reference. ECM: extracellular matrix, (LS)EC: (liver sinusoidal) endothelial cell, HEP: hepatocyte, HSC: hepatic stellate cell, MP: macrophage, NO: nitric oxide, PDGFbb: platelet-derived growth factor bb.

**Table S3 – Viability of NPF cell suspensions before and after cryopreservation in different media.** Human NPF from 2 donors (155 and 156) was cryopreserved in 4 different media in parallel. Table shows viability evaluated by Trypan blue exclusion (i) before cryopreservation, expressed as percentage of the total liver cell suspension (*) and (ii) after thawing, expressed as percentage of the viability before cryopreservation. DMEM: Dulbecco’s modified Eagle medium, DMSO: dimethylsulfoxide, FBS: fetal bovine serum, NPF: non-parenchymal fraction.

**Table S4 - Yields of human primary liver cells.** Yields of hepatocytes and non-parenchymal cells were extrapolated from the digested liver mass and from the viable cell counts using trypan blue exclusion. /: non-isolated fraction, NA^1^: missing liver weight, NA^2^: missing value of viable non-parenchymal cell isolated. (LS)EC: (liver sinusoidal) endothelial cells, HEP: hepatocyte, HSC: hepatic stellate cells, MP: macrophage.

**Table S5 - Yields of lyophilized HL-ECM.** Yields of human liver extracellular matrix (HL-ECM) were calculated as the mass ratio of the final product and of the decellularized liver mass.

**Table S6 – Extended proteomic data.**
