# Supplementary figures and images for: Human Liver-Derived Extracellular Matrix for the Culture of Distinct Human Primary Liver Cells
Source: Cells. 2020 May 30;9(6):1357. doi: 10.3390/cells9061357 (PMC7349413; doi:10.3390/cells9061357)

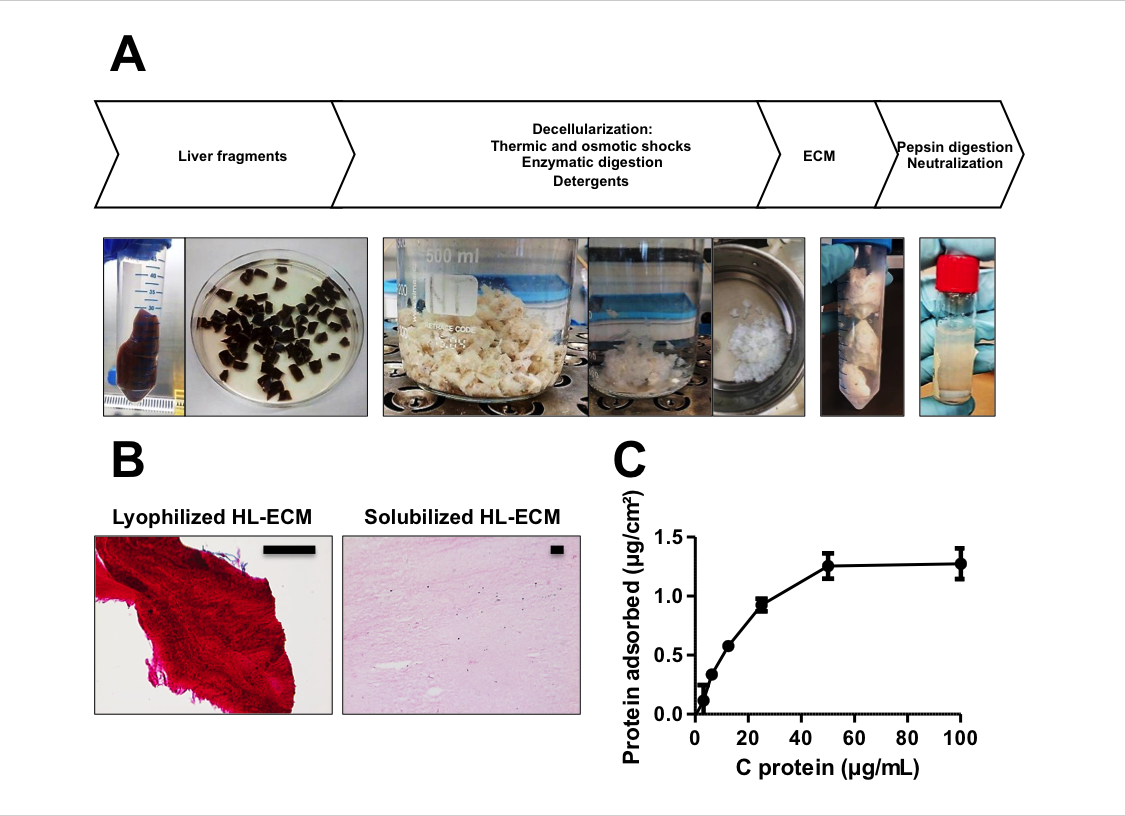

Supplement: Supplementary file 1 [file cells-09-01357-s001.zip › Figure S1_revised.tiff]

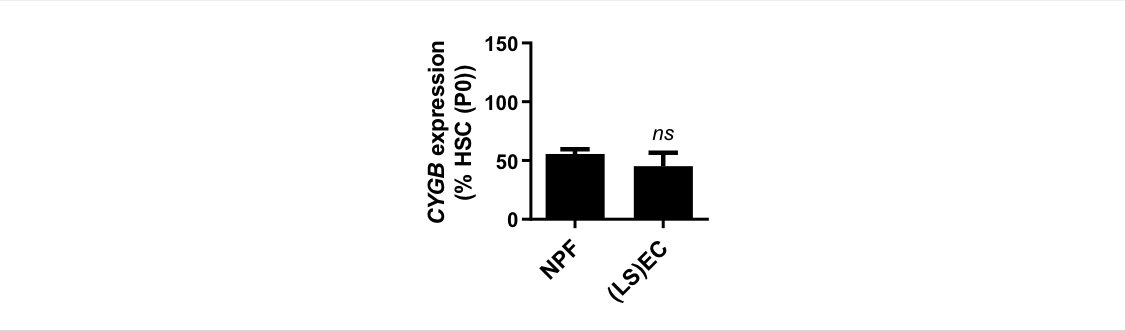

Supplement: Supplementary file 1 [file cells-09-01357-s001.zip › Figure S2_revised.tiff]

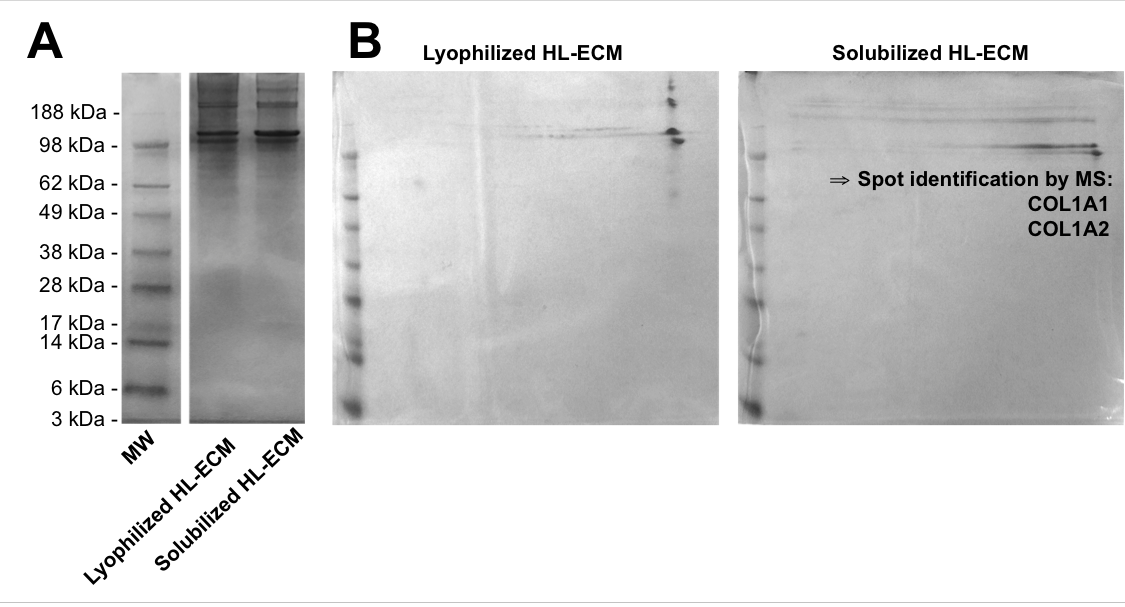

Supplement: Supplementary file 1 [file cells-09-01357-s001.zip › Figure S3_revised.tiff]

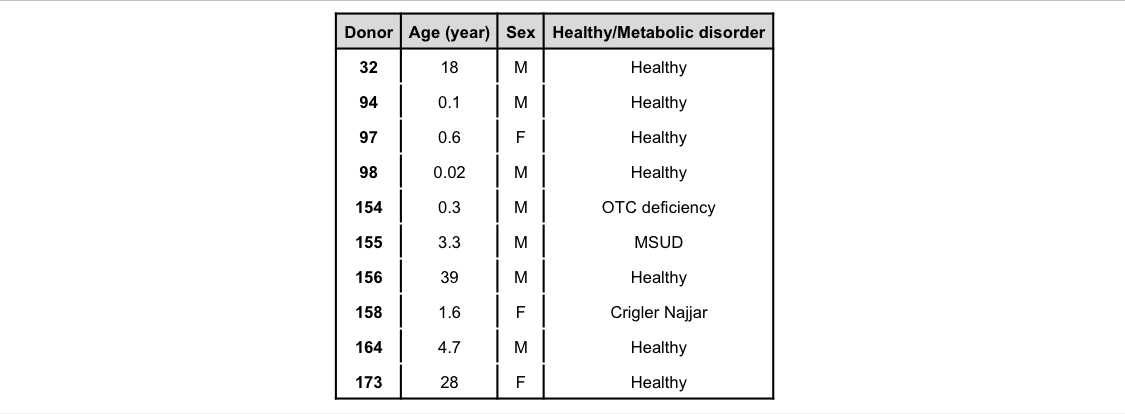

Supplement: Supplementary file 1 [file cells-09-01357-s001.zip › Table S1_revised.tiff]

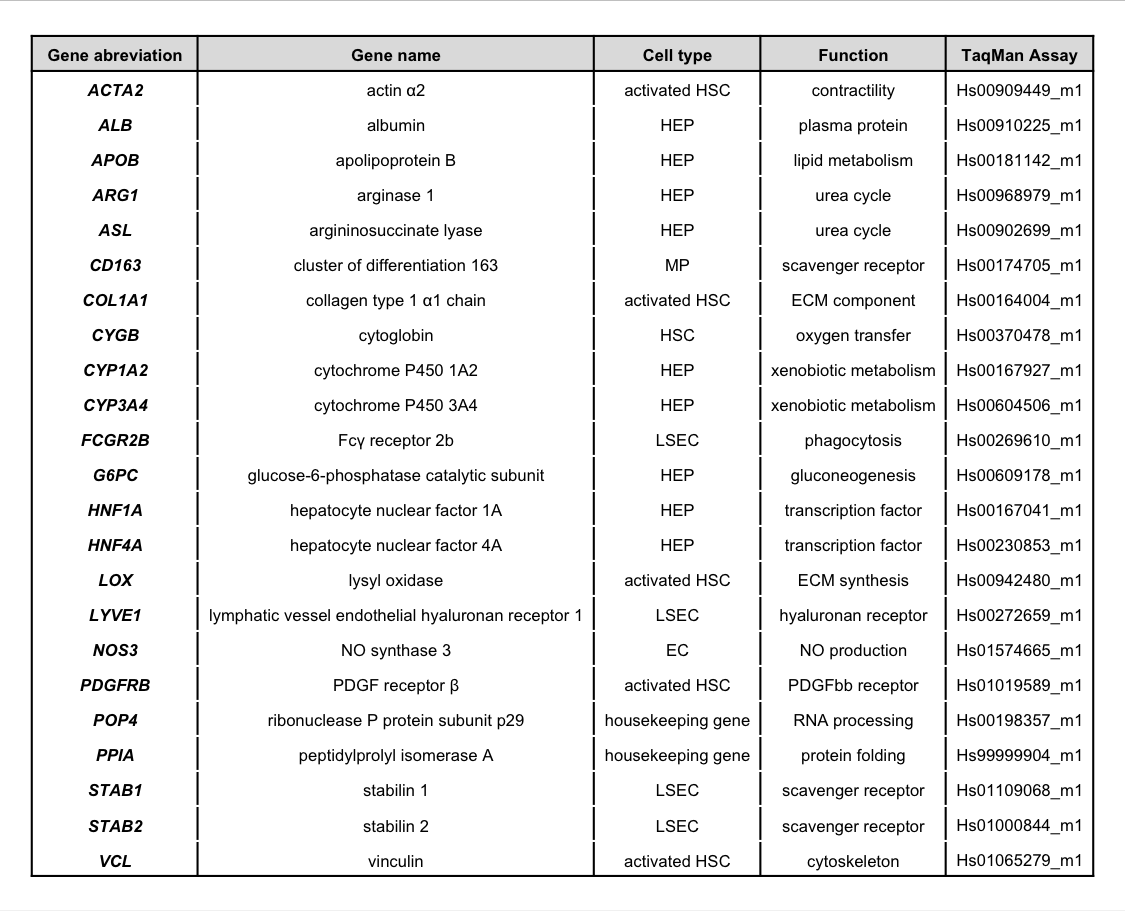

Supplement: Supplementary file 1 [file cells-09-01357-s001.zip › Table S2_revised.tiff]

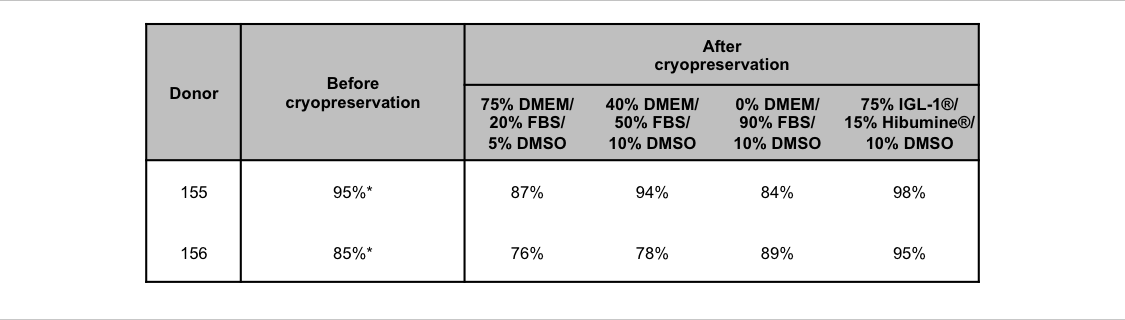

Supplement: Supplementary file 1 [file cells-09-01357-s001.zip › Table S3_revised.tiff]

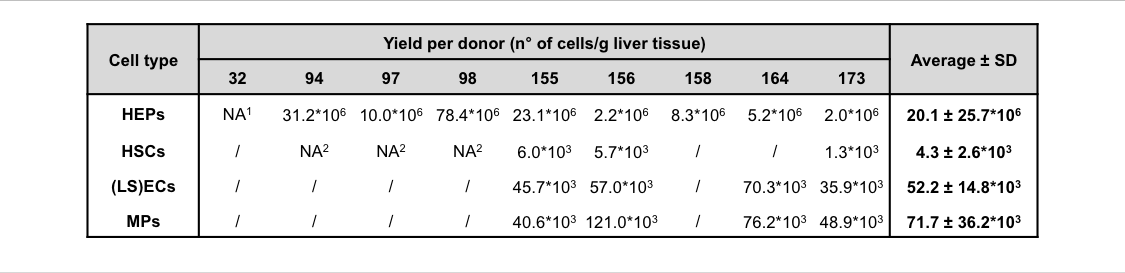

Supplement: Supplementary file 1 [file cells-09-01357-s001.zip › Table S4_revised.tiff]

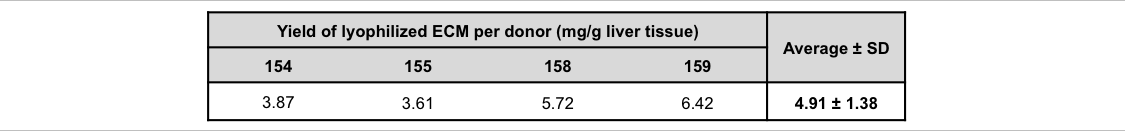

Supplement: Supplementary file 1 [file cells-09-01357-s001.zip › Table S5_revised.tiff]
